# Using Financial Incentives and Market Mechanisms to Improve Hospitals’ Performance; A Double-edged Sword

**DOI:** 10.34172/ijhpm.2023.8088

**Published:** 2023-05-21

**Authors:** Aidin Aryankhesal

**Affiliations:** School of Health Sciences, Faculty of Medicine and Health Sciences, University of East Anglia, Norwich, UK

## Dear Editor,

 Even with the improvement of public knowledge about healthcare and health services, the healthcare sector remains an incomplete market due to the complexity of health conditions as well as the adoption of high-tech products and devices in the health sector.^1^ Aware of such incompleteness, health sector policy-makers play their stewardship role through the so-called control knobs to regulate efficiency, quality, and access as intermediate performance measures^2^ (see Figure). Fulfilling such intermediate goals can assure people are in good health, satisfied with the care and services they receive, and protected from risks such as catastrophic expenses.

**Figure:**
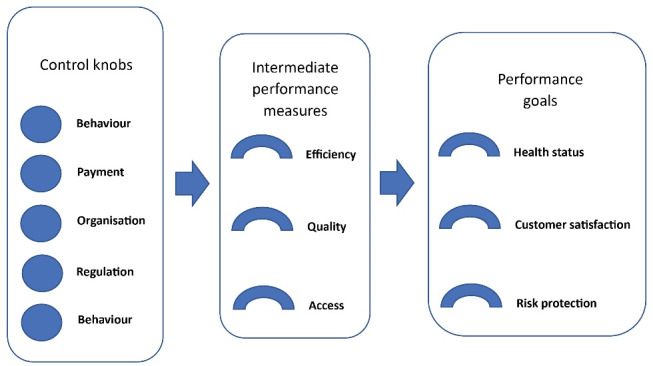
The Control Knobs for Health System Reforms.

 Two recent publications showed that performance-based financing (PBF) in Sub-Saharan Africa^3^ and licensing mechanisms in France,^4^ as direct and indirect financial mechanisms respectively, are developed to shape healthcare providers’ and hospitals’ behaviour and improve quality. As shown in Figure, financing and payments are two key items of the control knobs employed in the health sector for such reforms. Evidence also suggests that financial incentives are strong motives for triggering changes across healthcare providers,^5-8^ although such changes may not always result in quality improvement.^9^ Hence, due to such a strong impact, financial incentives can spread through health systems quickly.^3^ While the appropriate application of PBF and creating competition among providers for their share of the market through licensing mechanisms can be an attractive and influential means for policy-makers, such mechanisms should be used with caution.

 One important root of why PBF or licensing procedures may fail to reach the final goals of health systems lies in a lack of a comprehensive view when setting the intermediate performance measures and how they are defined and are in mutual relation with the final goals. An example of when final goals can be missed is when quality is defined as adopting new technologies, including advanced biomedical equipment or advanced procedures. While the adoption of new technologies can be defined as inputs and processes, its association with the final goals should be monitored by health policy-makers. Developing countries are good examples of importing extravagant high-tech biomedical equipment or procedures^10^ with limited effectiveness.^11,12^ Such adoptions, which are generally made with no need assessment,^13^ impose a high financial burden on governments and can result in induced demands and inappropriate indications because providers would like to see a return on their investments. Health technology assessment studies can address whether such technologies are needed. Moreover, high-tech procedures need advanced pieces of training before applying them which is sometimes missed in the settings that adopt such technologies.^10^

 Another possible consequence of using PBF and licensing, if the control knobs aspects are not considered carefully, can be limiting the public access to services. In such cases, the final goals are considered by policy-makers but the intermediate performance measures are neglected; the financial mechanisms can push smaller healthcare properties to merge with bigger ones due to the economy of scale rule.^4^ As mergers usually work towards the concentration of healthcare properties from the originally decentralised centres, access is generally affected. In some extreme scenarios, PBF can push hospitals into a vicious circle where poor-performing properties cannot improve their performance or even deteriorate since they receive a lower range of finance^14^ and may push to charge patients more.^15^ Such properties which are penalised for their performance, are in danger of bankruptcy and getting shut down which again limits public access to services.

 In summary, financial motivation mechanisms, including direct features such as PBF or indirect ones including licensing, can work as double-edged swords and should be considered with caution. When defining performance in the PBF mechanism, a comprehensive view should be applied so that all aspects of performance including intermediate and final goals are considered. The included intermediate and final goals should also be in rational mutual associations. The PBF and other financial motivation mechanisms should be applied in combination with other financing methods so that low-performing entities can survive the possible economic pressures, otherwise, people’s access to healthcare can be damaged.

## Ethical issues

 Not applicable.

## Competing interests

 Author declares that he has no competing interests.
